# Simultaneous SANS/FTIR measurement system incorporating the ATR sampling method

**DOI:** 10.1107/S1600576723007744

**Published:** 2023-09-27

**Authors:** Fumitoshi Kaneko, Aurel Radulescu, Hiroshi Nakagawa

**Affiliations:** a Graduate School of Science, Osaka University, 1-1 Machikaneyama, Toyonaka, Osaka 560-0043, Japan; b Jülich Centre for Neutron Science (JCNS), Heinz Maier-Leibnitz Zentrum (MLZ), 11 Lichtenbergstrasse 1, 85748 Garching, Germany; c Hierarchical Structure Research Group, Materials Sciences Research Center, Japan Atomic Energy Agency (JAEA), 2-4 Shirane, Shirakata, Tokai-mura, Naka-gun, Ibaraki 319-1195, Japan; d J-PARC Center, Japan Atomic Energy Agency (JAEA), 2-4 Shirane, Shirakata, Tokai-mura, Naka-gun, Ibaraki 319-1195, Japan; Tohoku University, Japan

**Keywords:** small-angle neutron scattering, SANS, Fourier transform infrared spectroscopy, FTIR, simultaneous measurement, attenuated total reflectance, ATR

## Abstract

Small-angle neutron scattering (SANS) is widely used as a powerful technique to study the higher-order structure of soft matter. To increase the reliability of SANS profile analysis for complex multi-component systems, combining different types of structural information obtained by other methods is desirable. A simultaneous measurement system combining SANS and Fourier transform infrared (FTIR) spectroscopy meets this objective. It is beneficial for targets where matching the timing of structural changes between experiments is difficult, but the issue is that samples suitable for SANS are too thick for the typical transmission FTIR method. To overcome this problem, a new simultaneous measurement system that employs the attenuated total reflectance (ART) sampling method for FTIR spectroscopy has been developed.

## Abstract 2

A simultaneous small-angle neutron scattering (SANS) and infrared absorption measurement system using the attenuated total reflection (ATF) sampling method for Fourier transform infrared (FTIR) spectroscopy has been developed to handle samples with large infrared absorption. This system allows each measurement to be performed under appropriate conditions.

## 1. Introduction

Small-angle neutron scattering (SANS) has been widely employed as an indispensable analytical tool for the higher-order structures of soft-matter systems (Roe, 2000 ▸; Higgins & Benoit, 1994 ▸). Combined with the partial deuteration technique, SANS can provide structural information focusing on a specific component of a multi-component system. On the other hand, since the SANS profile is exclusively determined by the scattering length density distribution in the sample system, we sometimes need some support to interpret the scattering profiles of complex multi-component systems. Obtaining other information about the soft-matter system would be helpful for a more reliable analysis. Vibrational spectroscopy is a good candidate for this purpose; it can provide a variety of information on each component within the measurement system, such as its concentration, molecular conformation and intermolecular interactions.

In research on soft-matter systems, it is sometimes difficult to control the time evolution of the structure within the object being measured. Changes over time in non-equilibrium states, such as structural changes and chemical reactions, may indicate accidental changes in state. Therefore, it can sometimes be difficult to map data obtained from different measurements to each other, even if the same sample is used for the measurement. In such a system, obtaining supporting information concurrently with neutron scattering measurements is desirable. Today, we have access to a number of Fourier transform infrared (FTIR) spectrometers, which are compact and demonstrate high performance suitable for time-resolved measurements. This advantage has led to attempts to combine FTIR with other techniques, such as X-ray and thermal measurements, for simultaneous measurements (Naylor *et al.*, 1995 ▸; Innocenzi *et al.*, 2007 ▸; Jaya Ratri & Tashiro, 2013 ▸; Mirabella, 1986 ▸; Pandita *et al.*, 2012 ▸). In previous studies, we developed a simultaneous measurement system of SANS and FTIR spectroscopy, which enabled the same target area to be investigated by both neutron scattering and IR transmission measurements (Kaneko *et al.*, 2015 ▸). We employed this methodology to study cocrystal systems of syndiotactic polystyrene (sPS), where sPS includes other chemical species as guest molecules in its crystalline region. This attempt allowed us to interpret the temperature-dependent SANS profiles in relation to the behavior of the guest molecules (Kaneko *et al.*, 2016 ▸, 2019 ▸). On the other hand, it has become clear that an essential issue needs to be addressed to apply this simultaneous SANS/FTIR measurement system to various targets.

SANS and FTIR each have their own sample requirements. The problem is that the appropriate sample thickness for SANS measurements is often too thick for FTIR transmission measurements; many IR bands are saturated, and only weak bands are available for study. Taking our studies on polymer cocrystals as an example, the bands attributed to the host polymer are saturated and, therefore, cannot be fully exploited. For this reason, we mainly use the information obtained from several IR bands of guest molecules. To overcome this drawback, we have turned our attention to the attenuated total reflectance (ATR) sampling method of FTIR spectroscopy.

The ATR sampling method, which employs the evanescent wave generated at the sample surface when IR radiation is totally reflected at an interface between the sample and a prism with a high refractive index, has been employed to measure the spectra of materials with very strong IR absorption and to perform surface analysis (Harrick, 1967 ▸; Knutson & Lyman, 1985 ▸; Milosevic, 2004 ▸). The ATR method effectively reduces the beam path length through the sample. The shorter effective path length improves the sensitivity of FTIR spectra because the sampling range from the surface to the penetration depth substantially contributes to the IR absorption. The penetration depth *d*_p_ corresponds to the depth at which the electric field of the evanescent wave decays to 1/*e* of that at the surface and is obtained by the following equation:

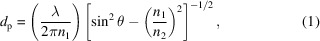

where λ is the wavelength, θ is the angle of incidence, and *n*_1_ and *n*_2_ are the refractive indices of the sample and the prism, respectively.

This paper reports our first attempt at simultaneous SANS/FTIR-ATR measurements. We will describe the experimental setup for the simultaneous measurements, together with some results of the test measurements on the gelation process of a polyethyl­ene glycol solution (Kobayashi & Kitagawa, 1997 ▸).

## 2. Instrumentation

### 2.1. SANS instrument

Simultaneous SANS/FTIR-ATR measurements were conducted using the focusing and polarized neutron ultra-small-angle scattering spectrometer (SANS-J-II) installed at the research reactor JRR-3 of the Japan Atomic Energy Agency (JAEA), Tokai, Japan. Monochromatic cold neutrons (wavelength λ = 0.65 nm and Δλ/λ = 15%) were provided by a velocity selector. The incident beam was defined using a 3.0 × 3.0 mm aperture 11 m upstream of the sample and an 8 mm-diameter aperture at the sample position. Scattered neutrons were detected by a two-dimensional ^3^He position-sensitive detector with a sensitive area of 65 × 65 cm^2^ (128 × 128 pixels). Using pinhole collimations, SANS-J-II covers the *Q* region from 0.03 to 1.2 nm^−1^ by changing the sample-to-detector distance (10 and 2.5 m), where *Q* is the magnitude of the scattering vector defined as *Q* = (4*π/λ*)sinθ with the scattering angle 2θ. The two-dimensional small-angle scattering obtained was corrected for detector sensitivity and instrument background scattering.

### 2.2. ATR sample holder

**Figure 1:**
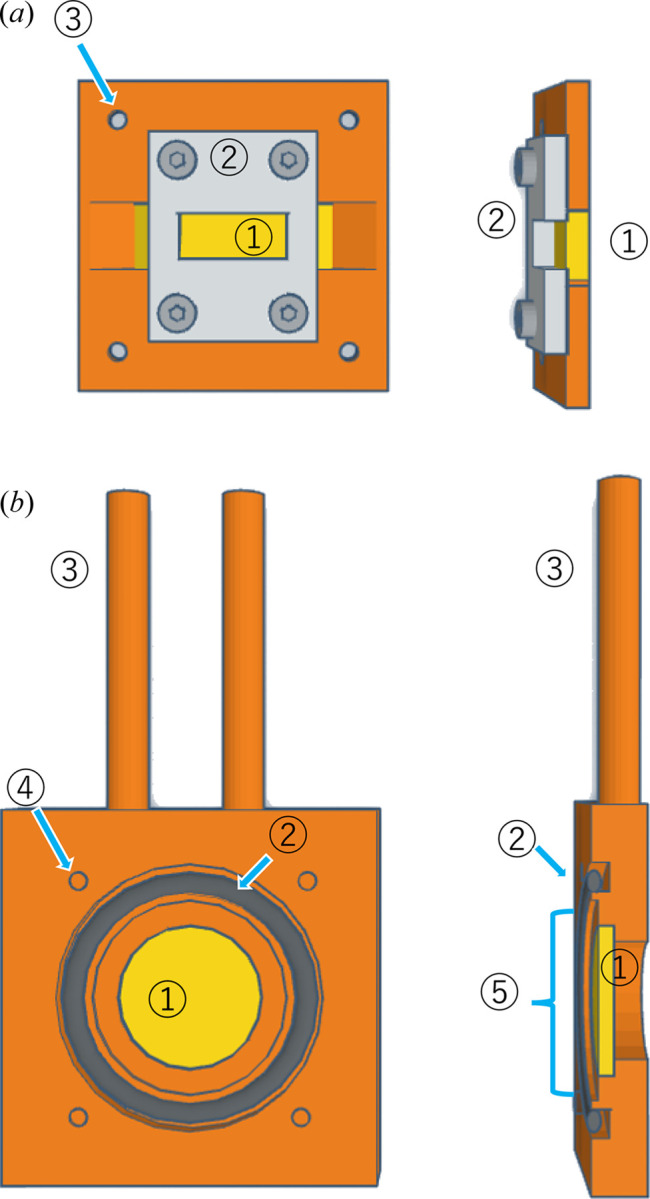
Front and cross-sectional views of the lid and body parts of the sample cell. (*a*) Lid with an integrated prism for the ATR sampling method. 1: ZnSe prism, 2: prism support plate, 3: through holes for lid–body coupling screws. (*b*) Body with a recess to accommodate the sample. 1: ZnSe plate, 2: O-ring, 3: pipes for heat-transfer fluid for temperature control, 4: threaded holes for lid–body coupling screws, 5: a recess to accommodate the sample. The ZnSe prism and the ZnSe plate are fixed to the lid and the body, respectively, using polyamide resin as adhesive material. The sample is held in a recess in the body section. The O-ring between the lid and the body ensures the tightness of this space.

Fig. 1 ▸ shows a schematic of the sample holder used in this study for simultaneous SANS/FTIR-ATR measurements. It is made of copper and divided into a lid and body parts. A trapezoidal ZnSe prism (base angle: 45°, length: 30 mm, width: 10 mm and thickness: 3 mm) for ATR measurements is attached to the lid. A circular ZnSe window with a diameter of 22 mm and a thickness of 2 mm is attached to the body. A circular depression 30 mm in diameter and 1 mm deep is provided over the ZnSe window to store samples. An O-ring between the body and the lid tightly seals the sample space. Liquid samples can be injected through a 1 mm-diameter hole in the side of the body. For temperature control, the interior of the body has a channel (cross-sectional area: 16 mm^2^) for circulating water along its edges.

### 2.3. Layout of equipment for simultaneous measurements

**Figure 2:**
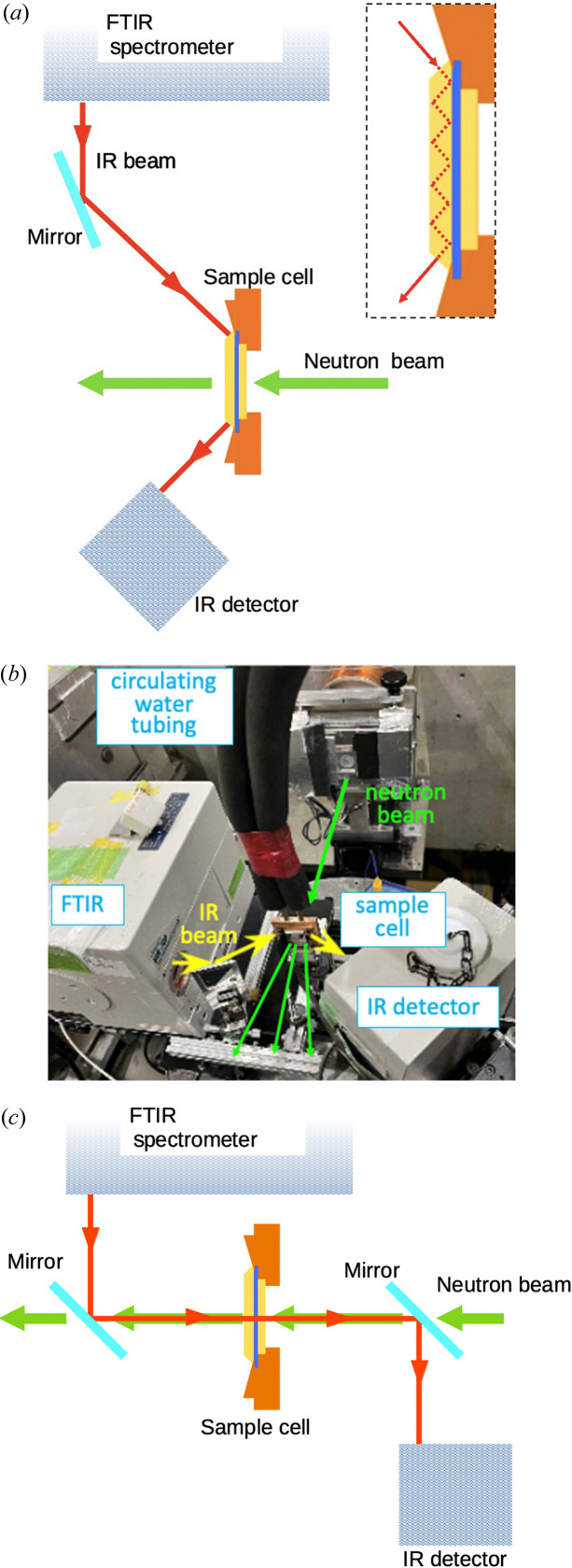
Equipment arrangement for simultaneous SANS/FTIR-ATR measurements. (*a*) Schematic of the optical arrangements for FTIR-ATR measurements. (*b*) Photograph of the on-site installation of the simultaneous SANS/FTIR measurement system. (*c*) Schematic of the optical arrangements for FTIR transmission measurements. The inset in (*a*) shows the light path in the prism.

An overview of the optical system used for simultaneous SANS/FTIR ATR measurements is schematically shown in Fig. 2 ▸(*a*), and the installation at the site is shown in Fig. 2 ▸(*b*). A combination of an FTIR spectrometer (VIR 200, JASCO Co.) and a wide-range mercury–cadmium–tellurium (MCT) detector (MCT-w, JASCO Co.) is employed; the detector is electrically connected to the FTIR main unit by a cable. The IR radiation emitted from the main unit, whose direction is adjusted by the mirror, is perpendicularly incident on the oblique side of the trapezoidal prism, is reflected five times on the sample surface at an incidence angle of 45° and exits through the other oblique side, toward the detector. The neutron beam is incident on the ZnSe circular window and passes through the sample chamber. The scattered neutrons from the sample pass through the ZnSe trapezoidal prism for ATR and arrive at the SANS-J-II spectrometer.

Simultaneous measurements with transmission IR spectroscopy are also possible by changing the IR optical path, as shown in Fig. 2 ▸(*c*). The two mirrors in the optical system are Al-coated quartz plates that transmit the neutron beam and reflect the IR beam. Owing to the characteristics of these mirrors, the IR beam is irradiated onto the sample coaxially with the neutron beam, and after passing through the sample, it is extracted and directed to the detector of the FTIR spectrometer.

## 3. Measurements

### 3.1. Evaluation sample

A deuterated tetra­hydro­furan (d-THF) solution of 5 wt% polyethyl­ene glycol (PEG) was prepared to test this simultaneous measurement system. The d-THF produced by EURISO-TOP has an isotope purity of 99.5% and the PEG sample supplied by JCNS has a molecular weight of approximately 2 × 10^4^ g mol^−1^ and is capped at both ends with methyl groups.

### 3.2. Simultaneous SANS/FTIR-ATR measurements

SANS 2D profile measurement conditions: 4 m sample-to-detector distance and 3 min intervals. FTIR-ATR spectral measurement conditions: 200 integrations, 4 cm^−1^ resolution and 3 min intervals.

## 4. Results

**Figure 3:**
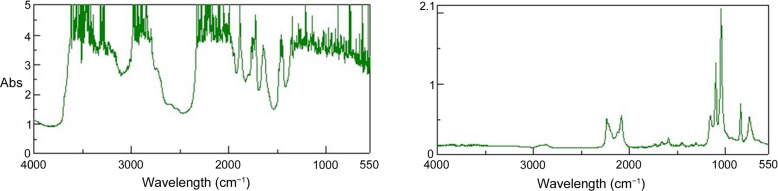
FTIR spectra of a d-THF solution containing 5 wt% PEG measured using the transmission method (left) and ATR method (right) at room temperature.

Fig. 3 ▸ compares the ATR and transmission spectra of a d-THF solution of 5 wt% PEG using the newly developed sample cell. The sample volume is designed to be 1 mm thick by 30 mm diameter, which is suitable for SANS measurements of solution samples, so most bands are saturated in the spectra measured by the transmission method. On the other hand, ATR measurements using this sample cell have yielded satisfactory spectra over the entire region. This is due to the characteristics of ATR spectroscopy, namely, the effective thickness of the sample is significantly thinner than in the case of the usual transmission measurement method. The estimated *d*_p_ values at 3000, 2000 and 1000 cm^−1^ are 0.6, 0.8 and 1.7 µm, respectively: the wavenumber dependence is reflected in the ATR spectra with enhanced absorption intensities in the lower-frequency region.

Reducing the sample thickness to obtain better IR transmission spectra can make it difficult to obtain analyzable SANS data. Therefore, the introduction of an ATR mode to the FTIR/SANS simultaneous measurement system is essential to handle a variety of samples flexibly.

**Figure 4:**
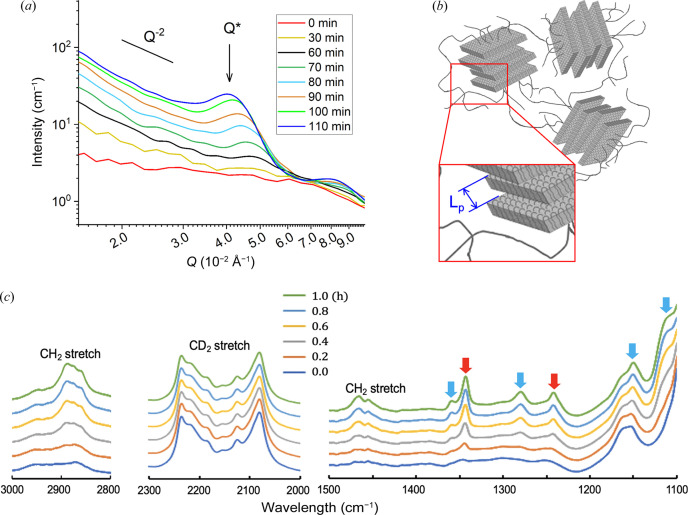
SANS profile and FTIR-ATR spectral changes of a d-THF solution of PEG: (*a*) time-dependent radially averaged SANS *I*(*Q*) profiles in the log–log plot; (*b*) the polymer morphology model; and (*c*) time-dependent IR spectral changes in the CH_2_ stretch region, CD_2_ stretch region and a portion of the fingerprint region, respectively. The bands with red and sky-blue arrows are attributed to the A_2_ and E_1_ modes, respectively.

The PEG solution transforms to a gel by cooling. Fig. 4 ▸ shows the one-dimensional SANS profile *I*(*Q*), a sketch of the morphology in the gel state as obtained from the interpretation of the SANS data and the FTIR-ATR spectral changes obtained using the simultaneous measurement system on cooling to 10.6°C.

When the sample reaches 10.6°C, the SANS scattering intensity increases continuously in the range *Q* < 1 × 10^−1^ Å^−1^ [Fig. 4 ▸(*a*)]. This is due to the crystallization of the PEG chains, and the polymer morphology evolves from the initial single-chain coil conformation in solution to stacked crystalline lamellae connected by long amorphous polymer segments [Fig. 4 ▸(*b*)]. A close examination of the temporal evolution of the scattering patterns confirms this simple scenario.

In the initial stage at 10.6°C, the typical scattering features of the polymer single coil can be observed in the scattering profile, namely a plateau at low *Q* and bending characteristic of the Guinier regime at about 0.06–0.07 Å^−1^, from which information about the coil radius of gyration can be obtained. A detailed characterization of poly(ethylene oxide) (PEO) chains in different solvents and for different chain ends, as performed by contrast variation SANS, can be found in a previous study where the influence of chain ends on PEO clustering in solution was also discussed (Hammouda *et al.*, 2004 ▸). The clustering strength in benzene and similar solvents is minimal for PEO where both end groups were –OCH_3_, as in the sample used in the current study.

With time, a continuous increase in intensity is observed at low *Q* with a stable characteristic power law of *Q* ^−2^ [Fig. 4 ▸(*a*)], indicating the formation of thin polymer lamellar structures (Hammouda, 2010 ▸). The lateral extent of the lamellae is too large for the length scale covered by the *Q* window used in the current experiment. This continuous increase in intensity toward low *Q* is accompanied by the development of an isotropic correlation peak at about *Q* = 5 × 10^−2^ Å^−1^ that appears after around 30 min of isothermal holding at 10.6°C and also grows continuously, shifting slightly toward the low-*Q* side. These results show that the lamellar structure already appears in the initial stage, and its amount increases monotonically, accompanied by a gradual increase of the lamellar spacing, *L*_p_ = 2π/Q* [Figs. 4 ▸(*a*) and 4 ▸(*b*)]. In the final stage of the gel, domains of stacked crystalline lamellae randomly oriented and interconnected by long amorphous polymer segments [Fig. 4 ▸(*b*)] and also containing amorphous interlamellar layers can be considered as the origin of such a scattering pattern. Similar scattering patterns were observed in the crystallization of polyethyl­ene from *o*-xylene (Wang, 2004 ▸) and in the crystallization of long-chain alkanes (waxes) in hydro­carbon solution driven by added polyethyl­ene-based diblock or random block copolymers (Radulescu *et al.*, 2007 ▸), where stacked crystalline lamellae in combination with amorphous interlamellar layers were characterized by contrast variation SANS.

Meanwhile, remarkable changes appear in the FTIR-ATR spectra, as shown in Fig. 4 ▸(*c*). The PEG component causes time-dependent spectral changes. The CH_2_ stretch and CH_2_ scissor bands around 2900 and 1460 cm^−1^ show significant intensity increases during the isothermal holding. The bands attributed to the IR-active A_2_ and E_1_ modes of the 7_2_ helix of polyethyl­ene glycol appear and increase in intensity with time (Yoshihara *et al.*, 1964 ▸; Matsuura & Miyazawa, 1968 ▸), some of which are given in Fig. 4 ▸(*c*). The bands at 1343 and 1242 cm^−1^ are attributed to the A_2_ modes, and those at 1359, 1280, 1150 and 1109 cm^−1^ are attributed to the E_1_ modes. These spectral changes suggest that the gradual conformational ordering of polyethyl­ene glycol from a random conformation to a 7_2_ helix conformation occurs during the isothermal holding (Takahashi & Tadokoro, 1973 ▸). On the other hand, the CD_2_ stretch region, attributable to the solvent d-THF, does not show any significant spectral changes, as shown in Fig. 4 ▸(*c*).

These observations obtained from the SANS and FTIR-ATR measurements indicate that the conformational ordering of polyethyl­ene glycol starts before the appearance of the lamellar reflection in the SANS profile, and the ratio of 7_2_ helices of polyethyl­ene glycol continuously increases during the period of measurements.

## 5. Discussion

Introduction of the ATR sampling method into the SANS/FTIR simultaneous measurement system enables the observation of all IR bands while ensuring appropriate sample thickness for SANS. It allows us to link two types of structural information: mesoscopic structures such as polymer higher-order structures recorded by SANS, and more localized structures such as molecular conformations recorded by FTIR. The combination of these two structural analysis methods has been very effective in tracking changes in the hierarchical structure of polymers over time.

The absorption intensity of ATR methods using evanescent waves is significantly lower than that of transmission methods. To compensate for this, ATR prisms that produce multiple total reflections at the sampling face are often employed. For bands with low absorption, the absorbance observed with *N*-time total reflections, *A_N_*, can be approximated as *N* *A*_1_, where *A*_1_ is the absorbance observed with one total reflection. Therefore, if appropriate absorption intensity cannot be obtained in the IR band observed, changing the number of total reflections at the ATR sampling plane is effective.

It should be considered that ATR spectra strongly reflect the structure in the vicinity of the sampling plane. The interaction between the ATR prism surface and the sample could significantly affect the structure formation near the prism–sample interface. In such cases, the structure of the area measured by the ATR method may not reflect the structure of the entire sample to be measured by neutron scattering. A preliminary comparison of transmission and ATR spectra may help to avoid this problem. Nevertheless, if the target system does not exhibit a particularly pronounced interaction with the ATR prism, the simultaneous measurement system can be an option for structural studies.
